# Supplementary figures and images for: Brainstem and Cortical Spreading Depolarization in a Closed Head Injury Rat Model
Source: Int J Mol Sci. 2021 Oct 28;22(21):11642. doi: 10.3390/ijms222111642 (PMC8584184; doi:10.3390/ijms222111642)

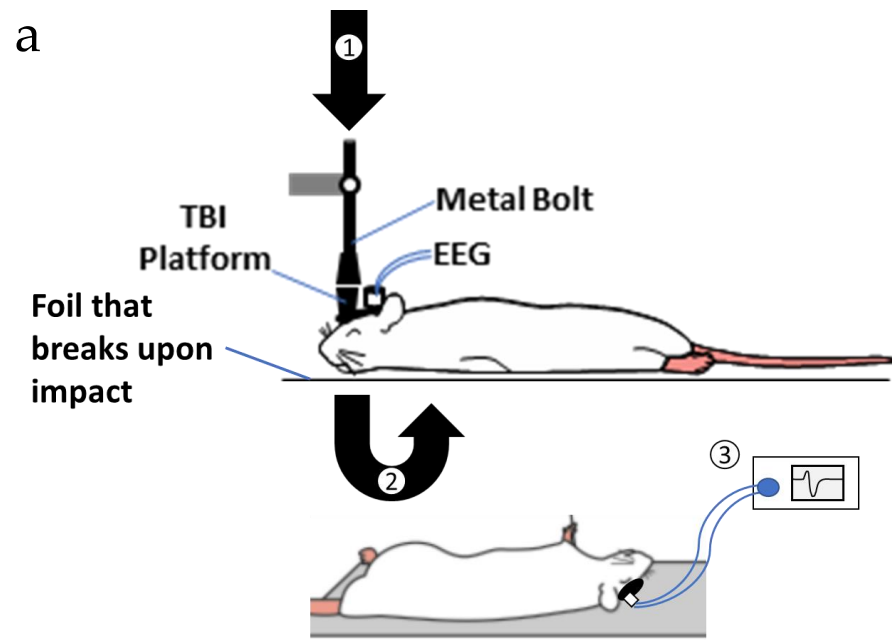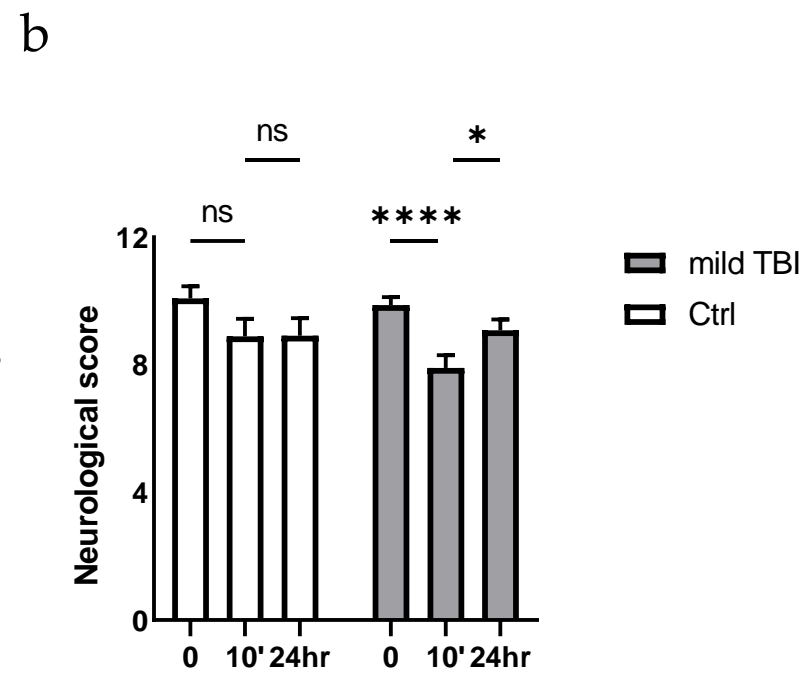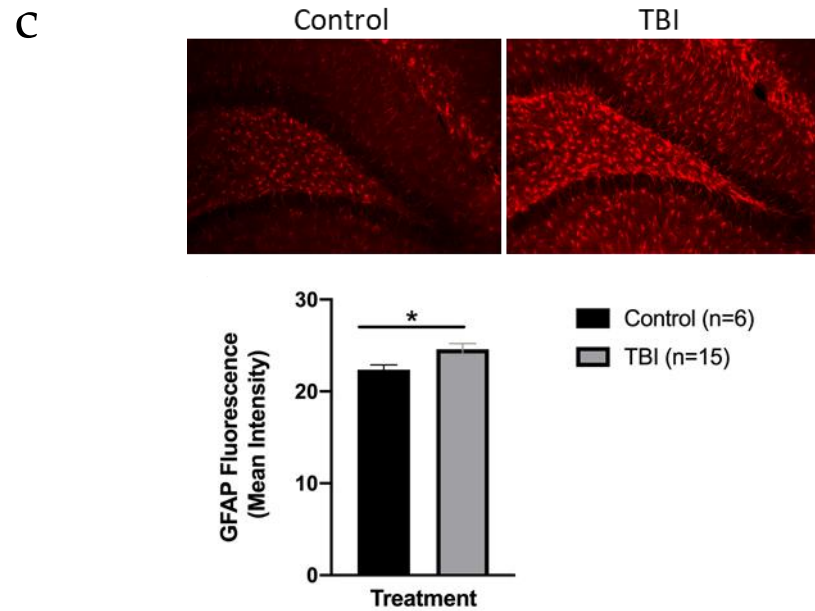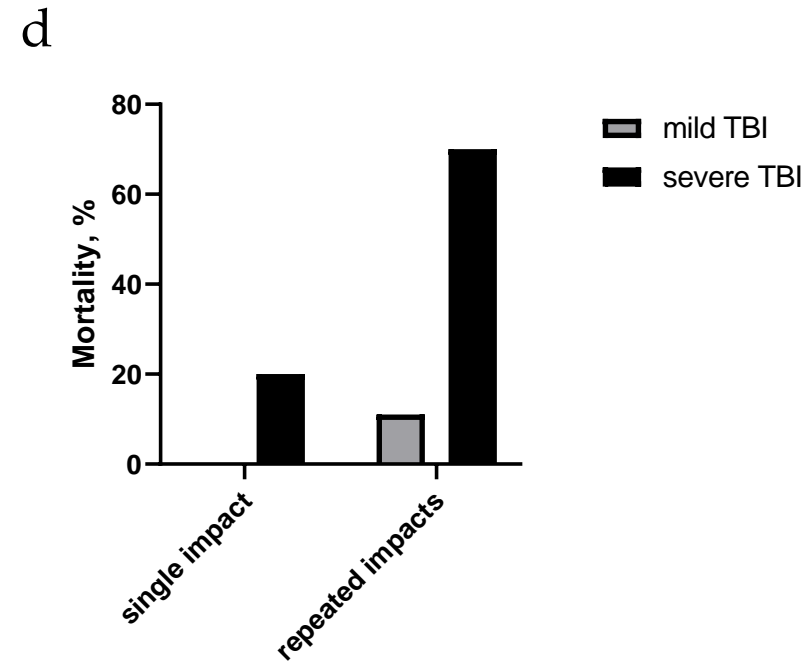

Supplement: Supplementary file 1 [file ijms-22-11642-s001.zip › Figure S1.pdf]

Antioxidants from brain homogenate

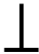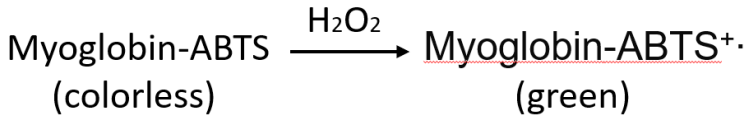

Supplement: Supplementary file 1 [file ijms-22-11642-s001.zip › Figure S2.pdf]
